# Prognostic Value of mNUTRIC and CONUT Scores in ICU Patients with Intracranial Hemorrhage

**DOI:** 10.3390/jcm15114022

**Published:** 2026-05-22

**Authors:** Mehtap Zengi, Gülbahar Çalışkan

**Affiliations:** Intensive Care Unit, Department of Anesthesiology and Reanimation, Bursa City Hospital, 16250 Bursa, Turkey; alkanbahar@yahoo.com

**Keywords:** intracranial hemorrhage, mNUTRIC score, CONUT score, intensive care unit

## Abstract

**Background**: Stroke is the most common neurological disorder in adults and a leading cause of mortality and disability. Intracerebral hemorrhage (ICH), although less frequent than ischemic stroke, is associated with higher morbidity and mortality and often requires ICU admission. Predicting mortality remains challenging due to disease heterogeneity. Objectives: This study evaluated the prognostic value of the modified Nutrition Risk in Critically Ill (mNUTRIC) and Controlling Nutritional Status (CONUT) scores, along with conventional severity scores (GCS, APACHE II, SOFA), in ICU patients with ICH. **Methods**: This retrospective cohort study included 347 ICU patients with ICH admitted between January 2019 and June 2025. Patients were stratified by survival status and nutritional and conventional severity scores were analyzed. Subgroup analysis was performed in patients with GCS ≤ 12 and APACHE II ≥ 17 (*n* = 96). Multivariate logistic regression and receiver operating characteristic (ROC) analyses assessed predictive performance. **Results**: ICU mortality was 24.2%. Deceased patients had lower GCS and higher APACHE II, SOFA, mNUTRIC, and CONUT scores (*p* < 0.001). Subgroup analysis showed higher mortality in patients with elevated mNUTRIC and CONUT scores (*p* = 0.038 and *p* = 0.005). Multivariate analysis identified GCS (OR = 0.675, *p* < 0.001) and CONUT (OR = 1.174, *p* = 0.040) as independent predictors; mNUTRIC was not significant. ROC analysis demonstrated good discrimination (AUC 0.818 for mNUTRIC and 0.81 for CONUT), with mNUTRIC being more specific and CONUT more sensitive. Optimal cut-off values were >3 for mNUTRIC and >4 for CONUT. **Conclusions**: Both mNUTRIC and CONUT scores are associated with mortality in ICU patients with ICH, with CONUT showing independent prognostic value. Their combined use may aid clinical decision-making.

## 1. Introduction

Stroke is the most common neurological disorder in adults worldwide and ranks first among neurological diseases, in terms of mortality and disability. Based on its etiopathogenesis, stroke is broadly classified into two main subtypes: ischemic and hemorrhagic. Although intracerebral hemorrhage (9–27%) is less common than ischemic stroke (73–81%), it is associated with significantly higher mortality and morbidity rates and is often considered a neurological emergency requiring admission to an intensive care unit [1].

Intracerebral hemorrhage may occur either as a result of trauma or spontaneously [1]. The most common causes of spontaneous intracerebral hemorrhage are hypertension and cerebral amyloid angiopathy, while secondary causes include vascular malformations, tumors and hemorrhagic transformation following thrombosis [2].

Patient prognosis is closely associated with age, comorbidities, hematoma size and intensive care scoring systems [3]. Therefore, various scoring systems reflecting disease severity have been developed to predict mortality and clinical outcomes in critically ill patients. The Glasgow Coma Scale (GCS) is widely used to assess neurological status, the Sequential Organ Failure Assessment (SOFA) score to evaluate organ failure and the Acute Physiology and Chronic Health Evaluation II (APACHE II) score to assess overall disease severity. In addition to these, the modified Nutrition Risk in Critically Ill (mNUTRIC) score, which evaluates nutritional status and stress response and the Controlling Nutritional Status (CONUT) score, which reflects immunonutritional status, have gained increasing importance in predicting clinical outcomes.

In recent years, international clinical nutrition guidelines, particularly those published by the European Society for Clinical Nutrition and Metabolism (ESPEN) and the American Society for Parenteral and Enteral Nutrition (ASPEN), have emphasized the critical role of early nutritional risk assessment in intensive care patients [4]. These guidelines recommend the routine use of validated nutritional screening tools such as the NUTRIC or mNUTRIC score in critically ill populations to identify patients who may benefit from aggressive nutritional support. Although these recommendations broadly apply to ICU populations, including patients with acute neurological conditions, there remains limited stroke-specific guidance, particularly for patients with intracerebral hemorrhage. Nevertheless, given the hypercatabolic state and inflammatory burden observed in these patients, nutritional assessment is considered clinically relevant and potentially prognostic.

The original NUTRIC score is calculated using parameters including age, number of comorbidities, length of hospital stay prior to intensive care unit admission, SOFA score, APACHE II score and Interleukin-6 (IL-6) levels [5]. However, because IL-6 cannot be routinely measured in all centers, the modified NUTRIC (mNUTRIC) score was developed by excluding this parameter. In the mNUTRIC scoring system, scores of 0–4 indicate low risk, whereas scores of 5–9 indicate high nutritional risk and are associated with increased mortality [6].

The CONUT score is an immunonutritional assessment tool calculated based on serum albumin concentration, total lymphocyte count and total cholesterol levels, reflecting not only nutritional status but also immune and metabolic reserves. Reductions in these parameters are associated with higher CONUT scores and poorer nutritional status. In the CONUT scoring system, scores of 0–4 are classified as low risk, while scores of 5–12 are considered high risk [7].

Despite their clinical utility, these scoring systems have certain limitations. Some parameters required for calculation, such as IL-6 in the original NUTRIC score or complete laboratory panels for CONUT, may not be readily available in all healthcare settings, particularly in resource-limited centers. Additionally, scores like APACHE II and SOFA require multiple physiological and laboratory variables, which may limit rapid bedside applicability. These constraints highlight the need for practical, accessible and reliable prognostic tools that can be universally implemented.

Unfortunately, there is no clear consensus in the literature regarding the superiority of these scoring systems in predicting clinical outcomes [8]. Therefore, this study aimed to compare the effectiveness of the mNUTRIC and CONUT scores in predicting mortality and clinical outcomes, in patients with intracerebral hemorrhage admitted to the intensive care unit and to evaluate their predictive performance in clinical practice.

## 2. Materials and Methods

This study was conducted as a single-center, retrospective cohort study with approval from the institutional ethics committee (Approval No.: 2025-12/14), between 1 January 2019 and 1 June 2025. All patients admitted to the intensive care unit with a diagnosis of intracerebral hemorrhage during the study period were included in the analysis. The requirement for informed consent was waived by the institutional ethics committee due to the retrospective design of the study and the use of anonymized data.

### 2.1. Inclusion Criteria

A total of 347 patients aged 18 years or older, with non-traumatic intracerebral hemorrhage confirmed by neuroimaging and who were monitored in the intensive care unit for at least 24 h, were included in the study.

### 2.2. Exclusion Criteria

Patients with traumatic intracerebral hemorrhage (*n* = 42), those under 18 years of age (*n* = 2), patients who died within 24 h of intensive care unit admission (*n* = 7) and patients with missing laboratory data required for calculating the scoring systems used in this study (GCS, APACHE II, SOFA, mNUTRIC, CONUT) (*n* = 9), were excluded from the analysis. Of the 9 patients, 4 had been transferred to another center within the first 24 h. The remaining 5 patients, who were followed during the pandemic period, could not be included in the analysis due to significantly incomplete laboratory and physical examination findings.

The patients’ demographic characteristics, comorbidities, treatment protocols, length of stay and discharge outcomes from the intensive care unit were retrospectively reviewed. Disease severity was assessed using the GCS, APACHE II, and SOFA scores, while nutritional status was evaluated using the mNUTRIC and CONUT scores, which were calculated within the first 24 h of ICU admission.

Patients were stratified into three groups based on their GCS scores: mild (15–13), moderate (12–9) and severe (≤8) [9]. According to the mNUTRIC score, patients scoring 0–4 were classified as low nutritional risk, whereas those scoring 5–9 were classified as high nutritional risk. Although the CONUT score is commonly categorized into four groups in the literature (0–1 normal, 2–4 low, 5–8 moderate, 9–12 high risk), it was consolidated into two categories (0–4: low risk, 5–12: high risk) for statistical analyses to ensure adequate group sizes and to allow clinically meaningful risk stratification [10,11]. For the subgroup analysis, patients with moderate or severe clinical conditions, defined as GCS ≤ 12 and APACHE II ≥ 17, were included [12].

### 2.3. Data Analysis

Data were analyzed using SPSS 20.0 (Statistical Package for the Social Sciences, SPSS Inc., Chicago, IL, USA). The normality of continuous variables was assessed with the Kolmogorov–Smirnov test. Normally distributed variables are presented as mean ± standard deviation, while non-normally distributed variables are presented as median (minimum–maximum). Categorical variables are expressed as counts and percentages. Comparisons between two groups according to mortality were performed using the Mann–Whitney U test for continuous variables and the Chi-square (χ^2^) test for categorical variables.

Independent predictors of mortality were first evaluated using univariable logistic regression. Subsequently, age, GCS, APACHE II, SOFA, mNUTRIC and CONUT scores were simultaneously included in a multivariable binary logistic regression, using the Enter method. Model significance was assessed with the Omnibus χ^2^ test, explanatory power with Nagelkerke R^2^, and model fit with the Hosmer–Lemeshow goodness-of-fit test. Odds ratios (ORs) and 95% confidence intervals (CIs) were calculated for each variable.

The predictive performance of mNUTRIC and CONUT scores for mortality was evaluated using Receiver Operating Characteristic (ROC) analysis. AUC and 95% CI were calculated, and the statistical significance of differences in AUC was tested using the DeLong method. Optimal cut-off values were determined by maximizing the Youden index (sensitivity + specificity − 1). At these thresholds, sensitivity, specificity, positive and negative predictive values (PPV/NPV), and positive and negative likelihood ratios (+LR/−LR) were calculated.

To assess multicollinearity among variables included in the multivariable model, Variance Inflation Factor (VIF) analysis was performed. Additionally, a post hoc power analysis was conducted for the subgroup analysis (*n* = 96; GCS ≤ 12 and APACHE II ≥ 17) using the chi-square test framework, with effect sizes expressed as Cohen’s w.

## 3. Results

**Figure 1:**
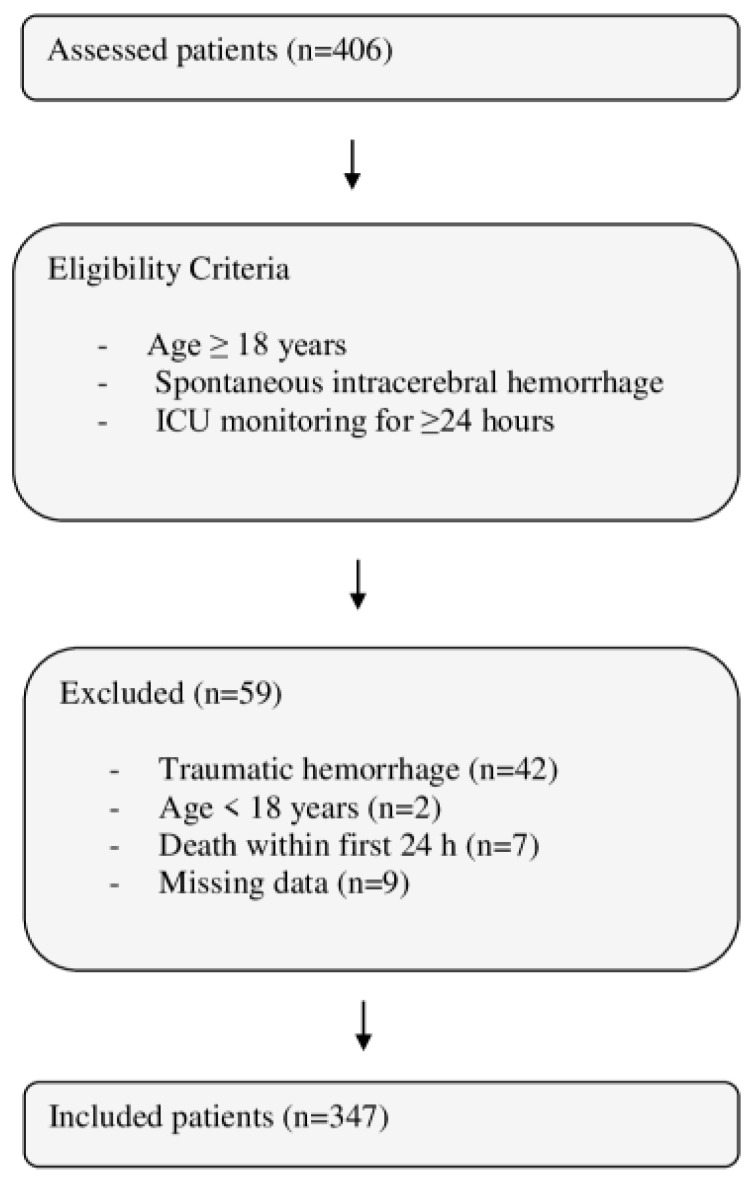
Patient selection flowchart. The arrows represent the sequential screening and inclusion process.

A total of 347 patients were included in the study (Figure 1). The mean age of the cohort was 62 years and 34.9% of the patients were female. Among the patients, 152 received medical treatment only, 59 received medical treatment combined with coil embolization, 127 underwent a surgical intervention and nine underwent both coil embolization and surgical procedures. Following these treatment protocols, intensive care unit mortality was 24.2% (*n* = 84). Of the 263 patients discharged from the ICU, 78.3% (*n* = 206) were transferred to the neurosurgery ward, while 21.7% (*n* = 57) were transferred to the palliative care unit.

Patients were divided into two groups based on mortality and compared (Table 1). The presence of additional systemic diseases (*p* = 0.026) and ICU length of stay (*p* = 0.035) differed significantly between the groups. No statistically significant differences were observed between the groups in terms of age and sex (*p* = 0.069 and *p* = 0.896, respectively) (Table 1).

The relationship between mortality and the scores was evaluated. GCS, APACHE II, and SOFA scores were significantly associated with mortality (*p* < 0.001). Both mNUTRIC and CONUT scores, analyzed as continuous variables and as low–high risk categories, also showed a significant association with mortality (*p* < 0.001).

A subgroup analysis was performed to evaluate the independent contribution of mNUTRIC and CONUT scores to mortality. To minimize potential confounding from low-risk patients, only 96 patients with moderate to severe clinical conditions (GCS ≤ 12 and APACHE II ≥ 17) were included. In this subgroup, mortality rates were significantly higher among patients in the high-risk categories of both mNUTRIC and CONUT scores (*p* = 0.038 and *p* = 0.005, respectively) (Table 2).

A multivariable binary logistic regression analysis (Enter method) was performed to identify independent predictors of mortality, including age, GCS, APACHE II, SOFA, mNUTRIC and CONUT scores. The model was statistically significant (Omnibus χ^2^ = 198.463, df = 6, *p* < 0.001) and demonstrated good explanatory power, with a Nagelkerke R^2^ of 0.651. According to the Hosmer–Lemeshow goodness-of-fit test, the model showed good calibration with the data (χ^2^ = 5.590, df = 8, *p* = 0.693). The overall classification accuracy of the model was 87.3%.

In the multivariable logistic regression analysis, GCS (OR = 0.675; 95% CI: 0.582–0.784; *p* < 0.001) and the CONUT score (OR = 1.174; 95% CI: 1.008–1.369; *p* = 0.040) were identified as independent predictors of mortality. Although the mNUTRIC score was significant in the univariable analysis, it did not retain statistical significance in the multivariable model (*p* = 0.217) (Table 3).

Based on these findings, the diagnostic performance of the mNUTRIC and CONUT scores in predicting mortality was evaluated using ROC analysis. The AUC was 0.818 for the mNUTRIC score (*p* < 0.001) and 0.81 for the CONUT score (*p* < 0.001). The optimal cut-off value for mNUTRIC was determined as >3, yielding a sensitivity of 69.8% and a specificity of 76.1%. For the CONUT score, the optimal cut-off value was >4, with a sensitivity of 87.9% and a specificity of 65.1% (Table 4).

**Figure 2:**
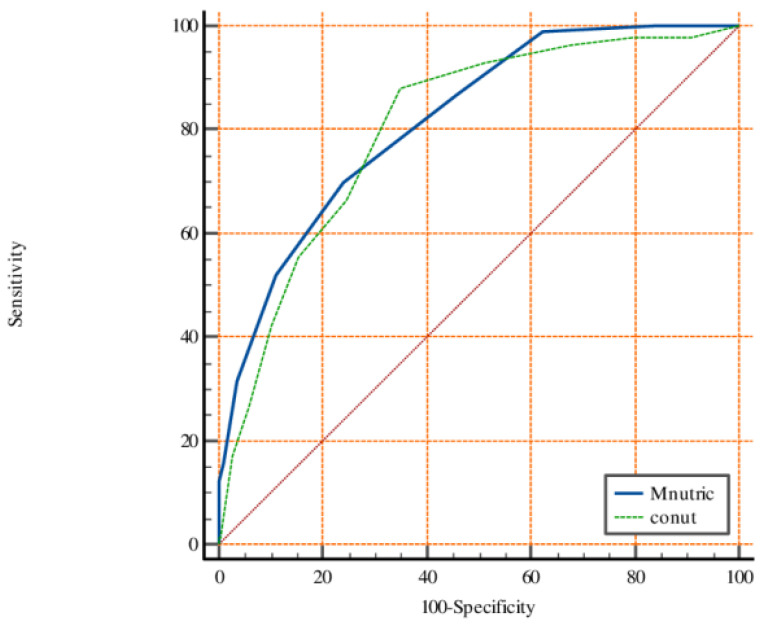
ROC Curve Analysis of mNUTRIC and CONUT Scores in Predicting Mortality. The diagonal red line represents the reference line (AUC = 0.50). A curve further from the reference line indicates better discriminative performance. The orange lines represent background grid lines included for visual reference only. mNUTRIC: modified Nutrition Risk in Critically Ill; CONUT: Controlling Nutritional Status; ROC: receiver operating characteristic; AUC: area under the curve.

The predictive performances of the mNUTRIC and CONUT scores for mortality were found to be similar, with both scores demonstrating good discriminative ability (AUCs of 0.818 and 0.81, respectively). No significant superiority was observed between the two measures in terms of AUC values (Figure 2).

## 4. Discussion

Predicting mortality in patients admitted to the intensive care unit due to intracranial hemorrhage is challenging because of the heterogeneous nature of the disease and the presence of multiple contributing factors. Therefore, the combined evaluation of parameters reflecting both disease severity and the patient’s metabolic and immune status is of clinical importance [13].

In this study, both conventional disease severity scores (GCS, APACHE II, SOFA) and nutrition-based indices (mNUTRIC and CONUT) were significantly associated with mortality, with the two nutritional scores demonstrating comparable discriminative performance.

When the findings were evaluated collectively, patients who died had significantly lower GCS scores and significantly higher APACHE II, SOFA, mNUTRIC and CONUT scores (*p* < 0.001). These results suggest that parameters reflecting both disease severity and nutritional status may be associated with mortality. In a study by Zhou et al. conducted in intensive care patients, APACHE II and SAPS II scores were reported to have high performance in predicting mortality [14]. Other studies have also highlighted APACHE II as a strong prognostic predictor of mortality [15,16]. Overall, these findings indicate that our results are generally consistent with previous literature.

From a nutritional perspective, the mNUTRIC score is an established tool for predicting mortality in critically ill patients. Studies by Balaban and Tong have reported that higher mNUTRIC scores are significantly associated with increased mortality risk [17,18]. Similarly, the CONUT score, which reflects immunonutritional status, has been shown to be closely associated with clinical outcomes, with higher scores linked to increased mortality. Studies by Yamaguchi et al. in geriatric patients and Bodolea et al. in patients with COVID-19 further support the prognostic value of nutritional risk scores across different patient groups [16,19].

In addition to these studies, several recent investigations have specifically explored the role of nutritional status as a predictor of mortality in ICU settings. Heyland et al. demonstrated that patients identified as high nutritional risk using the NUTRIC score had significantly improved outcomes when receiving adequate nutritional support, emphasizing the prognostic and therapeutic implications of nutritional assessment [5]. Similarly, studies focusing on neurocritical care populations have suggested that malnutrition is associated with prolonged ICU stay, increased complication rates and higher mortality. These findings reinforce the concept that nutritional status is not merely a background variable but a modifiable risk factor with direct clinical impact.

In parallel, further studies have strengthened the evidence supporting the prognostic role of nutritional status in critically ill patients. Rahman et al. demonstrated that higher mNUTRIC scores were independently associated with increased 28-day mortality, confirming its validity as a risk stratification tool in ICU populations [6]. Moreover, Almasaudi et al. reported that malnutrition assessed using validated nutritional indices was associated with higher mortality and prolonged hospitalization in ICU populations, further supporting the impact of nutritional status on clinical outcomes [20].

Beyond general ICU populations, emerging evidence in neurocritical care further supports these findings. Studies evaluating stroke and intracranial hemorrhage patients have shown that poor nutritional status is associated with unfavorable neurological outcomes, increased infection rates and higher mortality. In addition, research in stroke cohorts has demonstrated that malnutrition is linked to worse functional recovery and increased mortality. These findings suggest that early identification of nutritional risk may provide an opportunity for targeted interventions that could potentially improve both survival and functional outcomes.

Taken together, the growing body of literature indicates that nutritional assessment tools such as mNUTRIC and CONUT are not only complementary to traditional severity scores but may also provide independent and clinically actionable prognostic information in critically ill populations. This expanding evidence base highlights the growing recognition of nutritional status as a key determinant of clinical outcomes.

In the literature, studies directly comparing mNUTRIC and CONUT scores within the same population of intensive care patients with intracranial hemorrhage are limited, enhancing the novelty of our study. Therefore, a subgroup analysis was performed to more clearly assess the prognostic value of both nutritional scores. In this analysis, a more homogeneous patient population with comparable levels of neurological injury and systemic disease severity was defined by including patients with GCS ≤ 12 and APACHE II ≥ 17 [12]. This approach aimed to minimize the potential confounding effects of key disease severity determinants and to better delineate the contribution of nutrition-based scores to mortality. The analysis showed that mortality rates were significantly higher in patients with elevated mNUTRIC and CONUT scores (*p* = 0.038 and *p* = 0.005, respectively). These findings suggest that nutritional and immunological status may independently contribute to mortality, even among patients with similar disease severity.

In the multivariate analysis, the identification of GCS and CONUT scores as independent predictors of mortality confirms the central role of neurological, nutritional and immunological status in determining prognosis. Consistent with the literature, GCS has been established as a strong prognostic indicator, while the CONUT score has been shown to independently predict mortality [19]. In contrast, the loss of significance of the mNUTRIC score in the multivariate model may be attributed to its overlap with its components, namely APACHE II and SOFA; similarly, some studies have reported that mNUTRIC is not an independent predictor [17]. These findings indicate that, within the same model, the CONUT score provides a more independent and robust prognostic value. To assess multicollinearity among variables included in the multivariable logistic regression model, VIF analysis was performed. The mNUTRIC score showed a VIF of 11.35, indicating substantial collinearity with APACHE II (r = 0.722) and SOFA (r = 0.670), consistent with its composite nature. Age also demonstrated a high VIF (23.67), likely reflecting its shared contribution to both the mNUTRIC and APACHE II scores. In contrast, the CONUT score showed a VIF of 5.50, suggesting relatively lower collinearity and supporting its role as a more independent predictor in the multivariable model. These findings provide a statistical basis for the observed loss of significance of mNUTRIC in multivariable analysis.

ROC analysis including all patients showed that both mNUTRIC and CONUT scores had similar predictive performance for mortality, with high discriminative ability (AUCs of 0.818 and 0.81, respectively). The mNUTRIC score demonstrated higher specificity (76.1%) and positive likelihood ratio (+LR = 2.93), whereas the CONUT score exhibited higher sensitivity (87.9%) and superior negative likelihood ratio (−LR = 0.18). Accordingly, the combined use of these scores may offer complementary information in clinical practice.

However, despite their usefulness, these scoring systems are not without limitations. The mNUTRIC score, although practical, is partly derived from other severity indices such as APACHE II and SOFA, which may lead to collinearity and reduced independence in multivariate analyses. On the other hand, the CONUT score relies on laboratory parameters that can be influenced by acute-phase responses, fluid shifts and underlying metabolic conditions, potentially affecting its accuracy in critically ill patients. Furthermore, variability in laboratory availability and differences in institutional protocols may limit the universal applicability of these tools across different healthcare settings.

Moreover, as the scores assess different components—mNUTRIC reflecting disease severity and inflammation, and CONUT reflecting immunonutritional status—they can provide complementary prognostic insights when applied together.

In our study, ROC analysis identified the optimal cut-off values for predicting mortality as >3 for mNUTRIC and >4 for CONUT. In the literature, cut-off values are generally reported as 4–6 for mNUTRIC and ≥5–6 for CONUT [10,21]. However, data on these thresholds in intensive care patients with intracranial hemorrhage remain limited. Furthermore, to further explore the combined prognostic utility of both scores, a combined analysis using these dichotomized thresholds (mNUTRIC > 3 and CONUT > 4) was performed, and patients meeting both criteria demonstrated the highest discriminative performance for mortality prediction compared to either score alone. This combined approach enhanced overall risk stratification by integrating the high sensitivity of CONUT with the higher specificity of mNUTRIC, thereby providing complementary prognostic information.

However, the use of the Youden index to determine optimal cut-off values, which equally weights sensitivity and specificity, may not fully reflect clinical priorities in critically ill ICU patients. In this population, minimizing false-negative results is particularly important, as failure to identify high-risk patients may delay timely nutritional or therapeutic interventions. Therefore, threshold selection strategies favoring higher sensitivity may be more appropriate in this setting. Notably, the CONUT score demonstrated relatively high sensitivity (87.9%) at the >4 cut-off, suggesting that it may serve as a useful early screening tool. These findings highlight the need to interpret statistically derived cut-off values within the context of clinical decision-making and patient safety.

This study has several limitations. Its retrospective design and single-center setting may limit the generalizability of the findings. In addition, the lack of subgroup analysis based on intracranial hemorrhage subtypes precluded a more detailed prognostic evaluation. Additionally, hematoma volume and location are well-established independent predictors of ICH mortality and were not available as covariates in this retrospective analysis; their absence may confound the associations observed between nutritional scores and outcomes. Variability in ICU management practices, nutritional support strategies, and patient characteristics may also affect external validity. Therefore, the proposed cut-off values (mNUTRIC > 3 and CONUT > 4) should be interpreted with caution, and prospective multicenter studies are required to validate these thresholds before clinical implementation. We acknowledge that the subgroup analysis (*n* = 96) may be underpowered for detecting small effect sizes, particularly for the mNUTRIC score. Post hoc power analysis revealed that the achieved power for the mNUTRIC comparison was approximately 54.6% (Cohen’s w = 0.21), and a minimum of 176 patients would be required to achieve 80% power at the observed effect size. For the CONUT score, the achieved power was 73.1% (Cohen’s w = 0.26). These findings have been acknowledged as a limitation of the subgroup analysis, and prospective studies with larger sample sizes are warranted to confirm these results. Since several components of the CONUT score are negative acute-phase reactants, repeated measurements beyond the acute phase of critical illness (e.g., 72–96 h after ICU admission) are needed to better distinguish true nutritional status from the acute stress response, and we propose this as a direction for future prospective studies.

## 5. Conclusions

The present study is among the few to directly compare classical disease severity scores (GCS, APACHE II, SOFA) with nutrition-based scores (mNUTRIC and CONUT) in the same patient population. While both mNUTRIC and CONUT scores demonstrated prognostic value in ICU patients with intracerebral hemorrhage, the CONUT score emerged as an independent predictor.

These scores should not be considered solely as baseline nutritional assessment tools but also as indicators of disease severity and the dynamic clinical course during intensive care follow-up. In this context, mNUTRIC reflects systemic physiological stress and overall disease severity, whereas CONUT reflects immunonutritional status. Therefore, their combined use may provide complementary and more comprehensive prognostic information, thereby improving risk stratification and supporting clinical decision-making in ICU patients with intracerebral hemorrhage.

## Figures and Tables

**Table 1:** Baseline Characteristics.

Parameters (*n* = 347)	Non Survivors (*n* = 84)	Survivors (*n* = 263)	*p* Value
Age (years) min–max (median)	18–91 (66)	19–93 (61)	0.069
Gender, *n* (%) Male	54 (64.3)	172 (65.4)	0.896
Additional systemic disease, *n* (%)			0.026
None	19 (22.6)	71 (27)	
Single	15 (17.9)	78 (29.7)	
Multiple	50 (59.5)	114 (43.3)	
ICU length of stay (days) min–max (median)	1–121 (11.5)	1–154 (10)	0.035
GCS min–max (median)	3–14 (4)	3–15 (14)	<0.001
APACHE II score min–max (median)	2–40 (18.5)	1–34(10)	<0.001
SOFA Score min–max (median)	2–12 (7)	0–14 (3)	<0.001
mNUTRIC score min–max (median)	1–8 (5)	0–7 (2)	<0.001
mNUTRIC score category, *n* (%)			<0.001
Low (≤4)	42 (50)	235 (89.4)	
High (≥5)	42 (50)	28 (10.6)	
CONUT score min–max (median)	0–17 (7)	0–12 (4)	<0.001
CONUT score category, *n* (%)			<0.001
Normal/low	12 (14.3)	167 (63.5)	
Moderate/severe	72 (85.7)	96 (36.5)	

GCS: Glasgow Coma Scale; APACHE II: Acute Physiology and Chronic Health Evaluation II; SOFA: Sequential Organ Failure Assessment; mNUTRIC: modified Nutrition Risk in Critically Ill; CONUT: Controlling Nutritional Status; ICU: intensive care unit.

**Table 2:** Subgroup Mortality According to mNUTRIC and CONUT Score Categories.

Scores (*n* = 96)	Non Survivors (*n* = 59)	Survivors (*n* = 37)	*p* Value
mNutric score category, *n* (%)			*p* = 0.038
Low	22 (37.3)	22 (59.5)	
High	37 (62.7)	15 (40.5)	
CONUT score category, *n* (%)			*p* = 0.005
Normal/low	5 (8.5)	12 (32.4)	
Moderate/severe	54 (91.5)	25 (67.6)	

mNUTRIC: modified Nutrition Risk in Critically Ill; CONUT: Controlling Nutritional Status.

**Table 3:** Independent Predictors of Mortality: Multivariable Logistic Regression Analysis.

Parameters	B	S.E.	Wald	df	*p*	Exp (B)	95% CI Low	95% CI Upper
Age	−0.007	0.017	0.146	1	0.703	0.993	0.961	1.028
GCS	−0.393	0.076	26,667	1	<0.001	0.675	0.582	0.784
APACHE II score	−0.003	0.043	0.004	1	0.952	0.997	0.916	1.086
SOFA score	0.190	0.117	2.653	1	0.103	1.209	0.962	1.520
mNUTRIC score	0.254	0.206	1.523	1	0.217	1.289	0.861	1.928
CONUT score	0.161	0.078	4.236	1	0.040	1.174	1.008	1.369
Constant	−0.457	1.458	0.098	1	0.754	0.633		

B: regression coefficient; S.E.: standard error; df: degrees of freedom; Exp (B): odds ratio; CI: confidence interval. Dependent variable: Mortality; Nagelkerke R^2^ = 0.651.

**Table 4:** Diagnostic Performance of mNUTRIC and CONUT Scores in Predicting Mortality.

	AUC	*p*-Value	Cut-Off Value	Sensitivity (%)	95% CI	Specificity (%)	95% CI	+LR	−LR
mNutric	0.818	<0.001	>3	69.88	58.8–79.5	76.14	70.5–81.1	2.93	0.4
CONUT	0.81	<0.001	>4	87.95	79.0–941.	65.15	59.1–70.9	2.52	0.18

AUC: area under the curve; +LR: positive likelihood ratio; −LR: negative likelihood ratio; CI: confidence interval. PPV: positive predictive value; NPV: negative predictive value.

## Data Availability

The data presented in this study are available on request from the corresponding author due to patient privacy and confidentiality restrictions, in accordance with the requirements of the institutional ethics committee.
